# Duration of Pregnancy

**Published:** 1881-07-20

**Authors:** 


### *CORRESPONDENCE.*

---

### DURATION OF PREGNANCY.

---

DR. R. C. WORD, Atlanta, Georgia:

*Dear Doctor*—Will you be kind enough to give me the benefit of your knowledge on the subject I am about to mention. A young lady in this neighborhood, under promise of marriage, was seduced by a young man in August, 1880. Her menses appeared last on the 17th of August, and she was not confined until June 6th. According to our Obstetric Calendar her time (280 days), was out May 24, but she ran two weeks over time.

The parties have gone to law, the man taking comfort in the thought that when the *woman's* time was *out* so was *his*. He also pleads "not guilty" to the whole charge. As I will no doubt be called upon to testify in the case, I will be glad to hear from you.

Yours truly, C. J. B.

